# Australian and New Zealand nurses’ understanding and application of aseptic technique in clinical contexts: a cross-sectional mixed-method study

**DOI:** 10.1136/bmjoq-2025-003921

**Published:** 2026-06-30

**Authors:** Hannah M Kent, Joanne M Lewis, Sonja A Dawson, Brett G Mitchell

**Affiliations:** 1 School of Nursing and Health, Avondale University, Cooranbong, New South Wales, Australia; 2 School of Nursing and Midwifery, University of Technology Sydney, Sydney, New South Wales, Australia; 3 Central Coast Local Health District, Gosford Hospital, Gosford, New South Wales, Australia; 4 School of Nursing and Midwifery, Monash University, Melbourne, Victoria, Australia; 5 Hunter Medical Research Institute, Newcastle, New South Wales, Australia

**Keywords:** Aseptic technique, Asepsis, Nursing, Infection Prevention, Hospital-Acquired Infections, Education, Standard precautions

## Abstract

**Introduction:**

Aseptic technique remains fundamental in nursing practice and is essential in reducing hospital-acquired infections. Research shows that opportunities for nurses to update their knowledge and skills of aseptic technique are limited, and understanding of core principles can be inconsistent across healthcare and educational settings. Our aim was to capture the current understanding and application of aseptic technique among nurses in Australia and New Zealand.

**Method:**

We undertook a cross-sectional mixed-method survey design. Registered and enrolled nurses working in Australian or New Zealand healthcare or nursing education facilities were eligible to participate and were recruited between December 2024 and February 2025. Participants completed an anonymous online survey, informed by outcomes of a scoping review and adapted from existing surveys. Descriptive analysis was performed, with thematic analysis undertaken for open-ended questions.

**Results:**

In total 328 responses were received, primarily from Australia (n=275) and New Zealand (n=53). Respondents were predominantly from the public sector (62%), with 65% having more than 10 years of experience. While nearly all recognised the importance of aseptic technique for infection prevention, considerable variation was evident in how principles were described and applied. Terminology such as ‘clean’, ‘sterile’ and ‘aseptic’ was commonly misunderstood, and 48% of participants were unsure if their facility used a framework to guide aseptic technique. Less than half (47.2%) of nurses reported being reassessed on their aseptic technique skills in the past 5 years.

**Conclusions:**

These findings reveal variability in nurses’ understanding and practice of aseptic technique, highlighting opportunities for system-level improvement. Establishing standardised, evidence-based definitions and principles, supported by governance, may strengthen education, policy and clinical practice and promote patient safety. Further research is needed to reach a consensus on these definitions and principles.

**WHAT IS ALREADY KNOWN ON THIS TOPIC**

- Aseptic technique is a core infection prevention practice, yet international studies report inconsistencies in terminology, education and clinical application. This has not been adequately explored in the Australian and New Zealand context.

**WHAT THIS STUDY ADDS**

- This study identifies notable variability in Australian and New Zealand nurses’ understanding and practice of aseptic technique, including uncertainty around terminology, underlying principles, framework use and competency reassessment. These findings highlight opportunities for system-level improvement.

**HOW THIS STUDY MIGHT AFFECT RESEARCH, PRACTICE OR POLICY**

- The variability identified supports the need for greater standardisation of aseptic technique terminology, principles and expectations across policy, education and practice. Establishing consensus in these areas may enhance consistency in understanding and clinical application, thereby supporting patient safety.

## Introduction

 Healthcare-associated infections (HAIs) continue to pose a significant challenge worldwide, with 1 in 10 patients in large acute hospitals affected.^1^ Every year, an estimated 4.3 million patients in the European Union and European Economic Area, and approximately 165 000 individuals in Australia acquire a HAI.^1 2^ Common HAIs such as surgical site infections (SSIs), central line-associated bloodstream infections (CLABSIs) and catheter-associated urinary tract infections (CAUTIs) lead to increased healthcare costs, prolonged hospital admissions and higher patient morbidity and mortality.^3 4^ Enhancing infection prevention strategies through consistent clinical protocols, targeted staff education and alignment with best practice guidelines is crucial in reducing HAIs and combating antimicrobial resistance.^5^,^7^

Aseptic technique is a core component of infection prevention and is critical in preventing CAUTIs, SSIs and CLABSIs.^7 8^ The aim of aseptic technique is to minimise the risk of introducing harmful micro-organisms into wounds or vulnerable sites, while also preventing the transmission of pathogens from these areas to other individuals, including patients and healthcare staff.^9^ Nurses are integral to infection prevention as they are frequently responsible for performing aseptic technique across a wide range of clinical procedures.^10^ Aseptic technique is a required competency for initial nursing registration in both Australia and New Zealand, where it is taught and assessed within accredited undergraduate programmes that combine university-based learning with supervised clinical placements.^8 11 12^ While nurses must meet continuing professional development and competence requirements to maintain their registration, there is no national requirement for formal reassessment of specific clinical skills, such as aseptic technique, with ongoing education and competency maintenance largely managed at an organisational level. In the UK, studies have identified limited opportunities for nurses to update their knowledge or be reassessed in aseptic technique, revealing suboptimal understanding of asepsis and its principles.^13 14^ Gould *et al*^14^ and Issac *et al*^15^ reported challenges to aseptic technique in clinical settings, including a lack of understanding and standardisation in practice. Guidelines and literature regarding aseptic technique vary internationally, with a variety of terminology and approaches being used.^16 17^ This variation in terminology and principles does little to alleviate existing confusion in educational and clinical practice.^14 18^ Despite its importance, there is a paucity of evidence exploring how Australian and New Zealand nurses understand and apply aseptic technique in clinical settings. For clarity, the key terminology used in this study is outlined in (online supplemental table 1).

Informed by a recent scoping review,^17^ this study aimed to investigate nurses’ understanding and application of aseptic technique in clinical nursing settings. The specific objectives were to:

1. Assess the current level of knowledge of asepsis among nurses.
2. Examine how nurses have practised aseptic technique across different clinical contexts.
3. Identify perceptions and barriers experienced by clinical nurses when performing aseptic technique.
4. Determine the educational needs of nurses regarding asepsis and aseptic technique in the clinical setting.

By identifying potential gaps and variations in practice, this study will provide insights to inform policy refinement, support targeted education and guide further research, ultimately helping to reduce the risk of HAIs and promote patient safety.

## Methods

### Design

A cross-sectional online anonymous survey of registered nurses (RNs) and enrolled nurses (ENs) was conducted across Australia and New Zealand. This study was reported in accordance with the Strengthening the Reporting of Observational Studies in Epidemiology (STROBE) checklist for cross-sectional studies.^19^

## Participants

Eligible participants were RNs and ENs aged 18 years or older, currently working in an Australian or New Zealand healthcare or nursing education facility and registered with the Australian Health Practitioner Regulation Authority or the Nursing Council of New Zealand.

### Recruitment

Participants were recruited via a snowball approach between December 2024 and February 2025. Recruitment was conducted through professional associations, social media platforms (LinkedIn, Facebook), emails via publicly available contacts, personal and professional networks and announcements in hospital newsletters. The target sample size was 300 participants, consistent with response rates from previous published surveys on infection control practice in Australia and New Zealand.^20^,^22^ Informed consent was obtained online after participants read the participant information statement and selected ‘I agree to participate’. Participation was voluntary and anonymous, and no identifiable data linked to the survey was collected.

### Data collection

Data were collected using SurveyMonkey and settings were configured to prevent duplicate responses. The survey was informed by a scoping review of the literature and incorporated questions from existing surveys exploring aseptic technique among nurses.^13 14 17 23 24^ Excluding demographic items, the survey comprised 18 questions, some of which included multiple-item Likert-scale measures. Eight of these questions were directly adopted from previously published surveys developed by topic experts, six questions were adapted and the remaining four were newly developed based on the literature review.

The draft survey was piloted among a group of RNs and nursing academics prior to data collection, and minor modifications were made based on their feedback. The final survey consisted of four sections: demographic information, knowledge of asepsis, current practice, barriers to aseptic technique and educational needs. All data collected were stored securely in password-protected electronic files on the university’s internal server with two-factor authentication, accessed only by the research team and will be retained for 5 years before being permanently deleted.

### Data analysis

Data from the online survey were analysed using IBM SPSS V.29.0. Descriptive statistics were used to analyse survey responses. Subgroup analysis was not reported on, as the study was not powered for subgroup analysis. Qualitative responses for open-ended questions were analysed using content analysis, following the approach outlined by Elo and Kyngäs^25^ and Braun and Clarke’s^26^ thematic analysis techniques in NVivo (V.14).^27^ All submitted responses to each open-ended question were coded, with the number of responses reported at the top of each table. Data were coded by a single researcher; double coding was not undertaken. However, coding decisions and theme development were reviewed and discussed with the research team, and themes were refined through a consensus-based process. Data were checked prior to analysis, ensuring any identifiable information was removed.

## Results

### Demographics

A total of 328 participants consented to participate, with a survey completion rate of 75%. All participants who completed the survey answered every question, as responses were mandatory to progress. No imputations or assumptions for missing data were made. The 55 to 64 year age group represented the largest cohort (n=83, 25.3%), followed by 25 to 34 years (n=82, 25%). The majority of respondents were female (n=298, 90.9%). Participants were located across Australia and New Zealand, with the largest representation from New South Wales (n=121, 36.9%), followed by Victoria (n=64, 19.5%). Participants were drawn from both public and private hospitals and worked in a diverse range of clinical contexts.

Many participants had more than 20 years of experience in the nursing profession (n=136, 41.5%). The sample primarily consisted of RNs (n=142, 43.3%), followed by clinical nurse specialists (n=39, 11.9%) and clinical educators (n=34, 10.4%). Additionally, a notable number of respondents had undertaken further education beyond initial registration, commonly a postgraduate certificate (n=104, 31.7%) (table 1 and online supplemental table 2).

**Table 1:** Demographic details of participants (n=328)

Variable	n (%)
Participants’ characteristics	
**Age**	
18–24	19 (5.8)
25–34	82 (25)
35–44	65 (19.8)
45–54	67 (20.4)
55–64	83 (25.3)
65–74	12 (3.7)
**Gender**	
Female	298 (90.9)
Male	29 (8.8)
Prefer not to say	1 (0.3)
**Participant location**	
New South Wales	121 (36.9)
Queensland	24 (7.3)
Victoria	64 (19.5)
Tasmania	18 (5.5)
Australian Capital Territory	6 (1.8)
South Australia	8 (2.4)
Northern Territory	7 (2.1)
Western Australia	27 (8.2)
New Zealand*	53 (16.2)
**Participant hospital**	
Public	202 (61.6)
Private	90 (27.4)
Both	36 (11)
**Years of experience**	
<1	14 (4.3)
1–3	30 (9.1)
4–6	41 (12.5)
7–10	31 (9.5)
11–15	38 (11.6)
16–20	38 (11.6)
>20	136 (41.5)
**Participant profession**	
Enrolled nurse	13 (4)
Registered nurse	142 (43.3)
Clinical nurse specialist	39 (11.9)
Clinical nurse educator	34 (10.4)
Clinical nurse consultant	29 (8.8)
Nurse practitioner	3 (0.9)
Nurse manager/administrator	17 (5.2)
Nurse academic	42 (12.8)
Other	9 (2.7)
**Highest level of education**	
Diploma of nursing	20 (6.1)
Bachelor of nursing	91 (27.7)
Postgraduate certificate	104 (31.7)
Postgraduate diploma	14 (4.3)
Master of nursing	64 (19.5)
Masters’ (other)	10 (3.1)
PhD	21 (6.4)
Other	4 (1.2)

* North Island 33 (10.1) and South Island 20 (6.1).

### Current knowledge

Most participants agreed/strongly agreed (n=238, 94.1%) that aseptic technique was essential in preventing infections. Similarly, the majority agreed/strongly agreed (n=239, 94.5%) that correct aseptic technique is critical for patient safety (table 2). The majority of participants reported being confident when to apply aseptic technique to a nursing procedure (n=236, 93.4%) and that having a standardised approach to aseptic technique is important (n=239, 94.5%) (table 2). Content analysis of open-ended questions about participants’ understanding of aseptic technique revealed some conceptual understanding, but no consistent or shared definition. Common concepts included preventing contamination, protecting patients and staff and reducing microbial spread. Most responses focused on wound care. However, inconsistencies were evident around key terminology such as clean, sterile and aseptic (online supplemental table 5).

**Table 2:** Attitudes and beliefs of aseptic technique (n=253)

Topic	Strongly disagree *n (%*)	Disagree *n (%*)	Neutral *n (%*)	Agree *n (%*)	Strongly agree *n (%*)
I believe that aseptic technique is essential in preventing infections	15 (5.9)	–	–	9 (3.6)	229 (90.5)
I believe that correct aseptic technique is critical for patient safety	13 (5.1)	–	1 (0.4)	13 (5.1)	226 (89.4)
Attending an in-service and receiving updates on aseptic technique is a priority for me	10 (4)	18 (7.1)	23 (9.1)	98 (38.7)	104 (41.1)
Having a standardised approach to procedures requiring aseptic technique is important	9 (3.6)	2 (0.8)	3 (1.2)	46 (18.2)	193 (76.3)
I am confident when applying aseptic technique to a nursing procedure	8 (3.3)	2 (0.8)	6 (2.5)	43 (17)	193 (76.4)

### Difference in practice

Participants rated various elements of aseptic technique based on their importance when performing an aseptic procedure in their own practice. Hand hygiene was rated very important by 239 participants (94.5%), the highest of all principles (table 3). Maintenance of the aseptic field followed closely, with 236 (93.3%) participants rating it very important. Ratings for patient clothing were mixed, with 57 participants (22.5%) considering it very important and 37 (14.6%) unimportant. Similarly, for the number of people present, 68 participants (26.9%) rated it very important, while 33 (13%) viewed it as unimportant (table 3). Participants were asked in an open-ended question to list the principles of aseptic technique. Content analysis revealed similar results to the quantitative rating of some principles above. The most frequently mentioned principle was hand hygiene, followed by the importance of maintaining an aseptic field. Responses ranged from mentioning a single principle to listing over a dozen, often describing methods rather than principles. Terminology was inconsistent, with a lack of clarity between ‘sterile’, ‘aseptic’ and ‘clean’ fields (online supplemental table 6).

**Table 3:** Principles of aseptic technique (n=253)

Topic	Very unimportant *n (%*)	Unimportant *n (%*)	Neutral *n (%*)	Important *n (%*)	Very important *n (%*)
Sequencing to ensure the procedure is performed in a safe and appropriate order	1 (.4)	–	2 (.6)	53 (20.9)	197 (77.9)
Use of personal protective equipment	3 (1.2)	7 (2.8)	5 (2)	65 (25.7)	173 (68.4)
Environmental control	1 (.4)	1 (.4)	1 (.4)	71 (28.1)	179 (70.8)
Hand hygiene	3 (1.2)	–	1 (.3)	10 (4)	239 (94.5
Patient clothing	3 (.9)	37 (14.6)	34 (13.4)	122 (48.2)	57 (22.5)
Type of disinfectant used for cleaning surfaces	3 (1.2)	3 (1.2)	6 (2.4)	101 (39.9)	140 (55.3)
Patient education	4 (1.6)	1 (.4)	3 (1.2)	81 (32)	164 (64.8)
Maintenance of aseptic field	3 (1.2)	–	1 (.4)	13 (5.1)	236 (93.3)
Disposal of waste	2 (.8)	6 (2.4)	8 (3.2)	87 (34.4)	150 (59.3)
Speed of the procedure	13 (5.1)	71 (28.1)	34 (13.4)	105 (41.5)	30 (11.9)
Number of people present in the room	7 (2.8)	33 (13)	28 (11.1)	117 (46.2)	68 (26.9)

### Perception and barriers

The most frequently reported barrier to aseptic technique was lack of time (n=97), followed by variants in policy or procedure (n=88) and inadequate supplies or equipment (n=85) (table 4).

**Table 4:** What are the barriers to performing aseptic technique (n=238)

Topic*	n
Lack of time	97
Variants in policy or procedure	88
Inadequate supplies or equipment	85
Other†	75
Insufficient training or knowledge	70
Lack of support from colleagues or management	70

* Respondents could select multiple barriers.

† See Online supplemental table S8 for ‘other’ responses.

A total of 174 participants described experiencing or observing confusion around aseptic technique, with the majority of situations occurring in the ward setting. Through thematic analysis, five overarching themes emerged. These included variability in practice, lack of clarity around principles and terminology, confusion around clean, aseptic and sterile practice, knowledge gaps and environmental influences impacting practice. The most common misunderstandings were related to glove use, maintenance of the aseptic field and the distinction between clean, aseptic and sterile procedures (table 5).

**Table 5 T5:** Observation or experience of confusion surrounding aseptic technique (n=174)

Theme	Exemplars
**Variation in policies and practices** Differences between hospitals, wards and educational settings.	*“When a major hospital opened in WA, it borrowed policies from another government hospital … Each team believed their approach to aseptic technique was correct, leading to significant confusion.”* *“Variance in PICC procedures between local health districts.”* *“Different ways of practice from change in policy and variation between nursing staff.”* *“Changing protocols between facilities for the same procedure (e.g. removal of arterial lines), one required “sterile” conditions, the other a non-touch clean technique,”* *“Students see something different on placement to what we teach.”* *“Scrubbing IV cannula’s hubs, different times and ways of doing. Whether to use gloves for preparing IV fluids, different views on. Often no clear research one way or the other.”* *“In uni, I was taught one way, staff on the ward did it another.”*
**Lack of clarity around principles and terminology** Responses revealed ambiguity in the terminology and principles that are used to guide practice.	*“Staff generally know how to manage aseptic technique, but do not apply/understand the language required. Sequencing can sometimes be poor, and occasional inappropriate PPE (glove) use”* *“Prior to becoming an IPC CNE, my knowledge of aseptic technique was extremely low … I still find it confusing with key sites. Should key sites include areas that are covered under a dressing?… There are still areas that are confusing- even for someone who feels very confident with aseptic technique”* *“It’s often confusing with terminology … clean, aseptic, sterile, different staff interpret them differently.”* *“Accessing ports/PICC lines is confusing in my organisation because domestic people say it is a sterile procedure and the organisation advises wearing sterile gloves.”* *“There is a misunderstanding around what is a key or critical part.”*
**Confusion around clean, aseptic and sterile practice** Participants highlighted ongoing uncertainty in distinguishing between sterile, clean and aseptic practices.	*“Confused whether the removal of the arterial line is a sterile or clean procedure, had to look up the policy guidelines to confirm.”* *“Commonly seen confusion in IV ABs preparation and administration about the need to use gloves or not.”* *“There is so much inconsistency, at times, [during aseptic technique] hand hygiene is pretty much not existent”* *“There are degrees of confusion as with CVC removal using standard vs aseptic gloves, policies might state standard gloves use is adequate for PICC removal ….”* *“I have seen staff unsure whether removing sutures should be done as a sterile or clean procedure.”* *“Many nurses unsure when to wear gloves”* *“Use of clean vs sterile gloves when changing a dressing.”* *“PICC dressing treated like a basic wound.”*
**Knowledge gaps** Observations of staff or students contaminating sterile and aseptic fields highlighted gaps in technique and understanding.	*“A student nurse in the Operating Theatres pointed to and touched an instrument on the sterile set up. When asked why, they stated ‘I washed my hands!’”* *“Staff members touching sterile field and dressing materials that came directly in contact with a pt.’s wound with non-sterile gloves.”* *“[I see confusion} all the time, the timing of hand hygiene and the use of non-sterile gloves impacting hand hygiene are the main issues”* *“Often people are confused when using sterile gloves and miss when they have contaminated them. When wearing sterile gloves, they consider themselves ’sterile’ and don't seem to have a keen awareness of when they contaminate these gloves and render them 'non-sterile’.”* *“Yes, colleague donning gloves and touching everything from tray to the patient and everything in between, breaking the sterile field.”* *“Staff with sterile gloves on touching nonsterile items thinking they're sterile due to the gloves being sterile. Drives me nuts!!”* *“Educator told me not to worry about key parts, just keep gloves on.”* *“Junior staff … quite often get confused about which areas they can touch and not touch when they have sterile gloves on.”*
**Environmental and situational influences impacting practice** Situational pressures such as emergencies, patient escalation, new equipment or time constraints affected adherence to aseptic technique.	*“During an emergency situation … Prior to being able to set up field properly/using correct PPE … another nurse removed the port with general gloves and general alcohol wipes.”* *“When there has been a change in equipment such as new dressing packs or dressing types, new brands or new types of dressings not used in a hospital from patients coming in from the community.”* *“Often when applying dressings in a mental health setting. If a patient is escalated any dressing being applied is better than no dressing.”* *“Emergency, urgent situations … Mostly people not appreciating the importance and taking shortcuts.”* *“Variations in compliance to perioperative aseptic technique is staff dependent, mainly due to time constraints, as well as procedure dependent (dependent on infection risk at site).”* *“During COVID … for intubation patients in ICU … During medical emergency for patients with known infection such as MRSA, VRE.”*

### Educational needs

Educational needs were identified with a majority of respondents (n=240, 94.9%) indicating that it was important to regularly reassess competency in aseptic technique. Less than half (n=117, 47.6%) reported having their aseptic technique reassessed through a competency-based assessment in the last 5 years, whereas (n=129, 52.4%) had not. Despite this, a high proportion (n=196, 79.7%) indicated they had updated their knowledge of aseptic technique in their own time (table 6). The resources commonly used were e-learning provided by the organisation, reviewing practice guidelines and accessing protocols within their organisation (online supplemental table 3 and online supplemental table 8). Just 44.7% (n=113) of respondents indicated that their facility uses a framework to guide procedures requiring aseptic technique, while a notable 48.2% (n=122) were unsure whether their facility used a framework to guide aseptic practice or not (table 6). The most common frameworks used to guide aseptic practice were ANTT^28^ (n=32) and local policy (n=21) (online supplemental table 7). Participants indicated they would find topics such as the importance of aseptic technique in infection prevention, underlying principles of aseptic technique and practical elements of aseptic technique the most beneficial in educational material (online supplemental table 4).

**Table 6:** Educational needs (n=253)

Topic	Yes *n (%*)	No *n (%*)
Does your facility currently use a framework to guide procedures requiring aseptic technique?	113 (44.7)	18 (7.1)*
Do you believe it is important to regularly reassess competency in aseptic technique?	240 (94.9)	13 (5.1)
Have you received additional training in aseptic technique since your initial nurse training? (n=246)	185 (75.2)	61 (24.8)
Have you been reassessed for aseptic technique since your initial nursing training? (n=246)	161 (65.4)	85 (34.6)
Have your aseptic technique skills been re-assessed through a competency-based assessment in the last 5 years? (n=246)	117 (47.6)	129 (52.4)
Have you updated your knowledge regarding aseptic technique in your own time? (n=246)	196 (79.7)	50 (20.3)

* Unsure responses= 122 (48.2).

## Discussion

**Figure 1:**
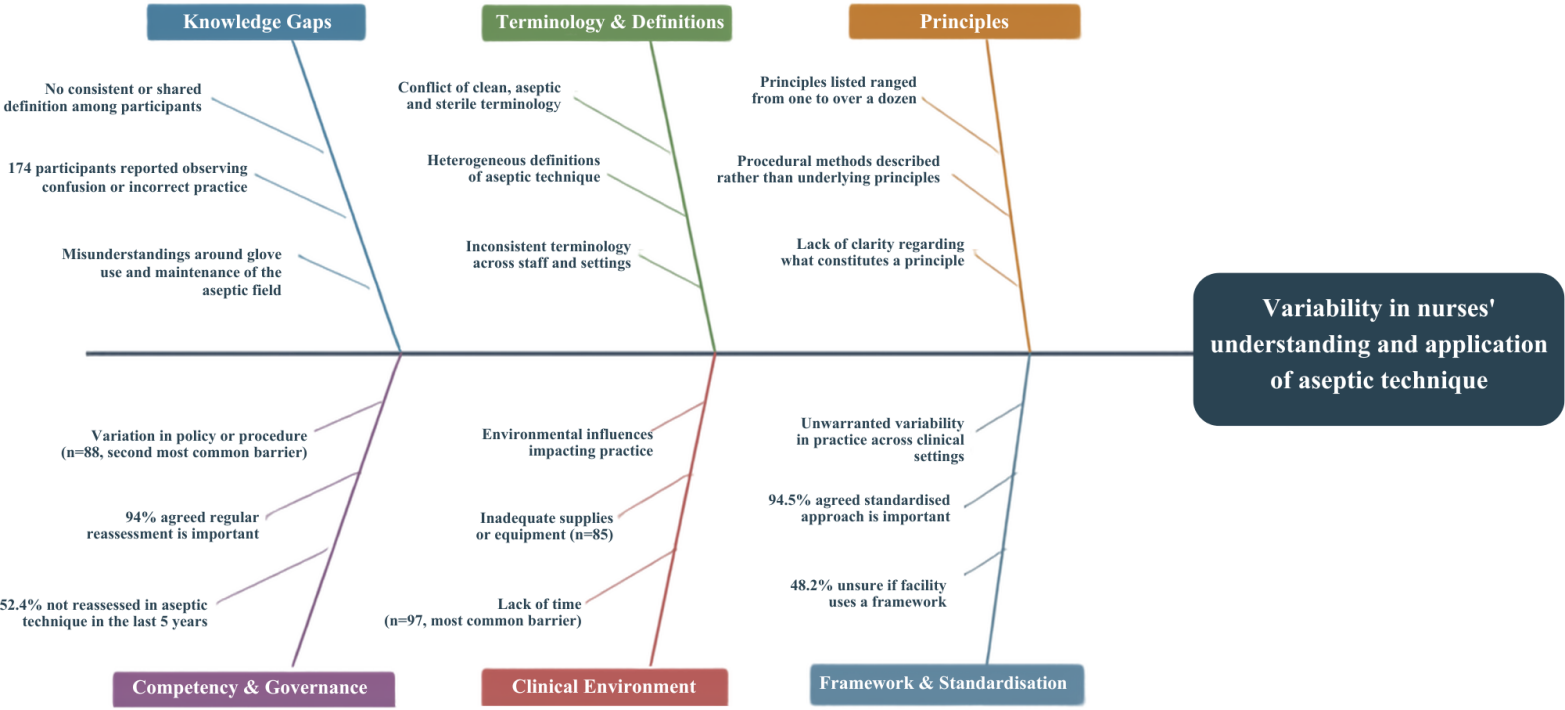
Proposed contributing factors to variability in nurses’ understanding and application of aseptic technique.

This cross-sectional study aimed to investigate nurses’ understanding and application of aseptic technique across Australia and New Zealand. To our knowledge, it is the first study in either country to capture nurses’ understanding and application of aseptic technique from a variety of clinical settings. Across the diverse clinical contexts, nurses recognised the importance of aseptic technique for infection prevention and patient safety, and reported confidence in applying aseptic technique (table 2). However, we found notable variation in their understanding of aseptic technique and its principles, suggesting potential knowledge gaps and unmet educational needs. From a quality improvement perspective, such inconsistency in understanding, terminology and application of aseptic technique is more likely to reflect unwarranted variation in a core patient safety practice rather than deliberate or appropriate contextual adaptation. The proposed contributory factors identified across the findings of this study are summarised in figure 1. These factors span multiple interrelated domains and are discussed further in the implications for improvement section.

Definitions of aseptic technique were relatively heterogeneous, with conflation of aseptic, sterile and clean terminology (online supplemental table 5). This aligns with the findings of Gould *et al*,^14^ who similarly reported a lack of clarity among UK nurses when asked to define aseptic technique. As noted by Flores^29^ and Aziz,^18^ unclear definitions in literature have the potential to influence variability in practice standards, which may in turn translate into variation in clinical practice.

Although participants rated some elements of aseptic technique in line with the principles outlined by the National Safety and Quality Health Standards (NSQHS)^8^ (table 3), many other elements not formally identified as principles were still rated as important or very important. This may suggest a lack of clarity regarding what constitutes a principle of aseptic technique. Several participants indicated patient education as a core principle, despite it not appearing as a principle in NSQHS guidelines.^6 8^ In contrast, New Zealand has recently developed their own framework with simplified principles, these are preparation, key parts and sites, and advice, with the final principle focusing on patient education.^30^ This difference in method, terminology and principles has also been identified across different international guidelines for aseptic technique.^16^ Hospital practice aims to align with evidence-based guidelines, but significant variability remains, highlighting the need for standardisation and improvement.^31^ These findings may suggest a system-level emphasis on aseptic technique as embedded within specific procedures rather than as a discrete competency, potentially limiting nurses’ ability to update their knowledge on best practice and complicating standardised education and competency assessment.

When participants were asked to list the principles of aseptic technique in an open-ended response, most participants identified at least one or more principle. There was notable variation in the principles described. The number of principles listed varied from one to over a dozen, and many participants described procedural methods rather than underlying principles. This variation is consistent with Hawker *et al*,^31^ who found considerable inconsistency in principles understood and taught by tertiary educators. There appears to be a focus on teaching procedural methods, most commonly in relation to wound care, rather than on the principles of aseptic technique that may be applied to a range of clinical procedures.^32^ Despite calls to emphasise principles in education,^14 33^ they remain poorly articulated, and the lack of consensus about what constitutes principles of aseptic technique further complicates teaching.^14 16^ This challenge is reflected in a national cross-sectional survey of 49 UK university institutions, which revealed considerable variation in the methods and principles used to teach aseptic technique.^24^ It is concerning that less than half of the participants in this study reported being re-assessed in aseptic technique in the last 5 years (table 6). The lack of routine reassessment highlights a governance gap in how aseptic technique competency is monitored, limiting organisations’ capacity to assure ongoing safe practice and to systematically identify areas for improvement.

While there has been development of non-touch technique frameworks, such as ANTT, if the core principles of aseptic technique are not well-defined or widely understood, it has the potential to cause confusion and inconsistency in the application of these frameworks and non-touch techniques. Australian Guidance does not specifically endorse ANTT; however, it recognises ANTT as a best-practice example of a clinical practice and education framework for aseptic technique.^6 34^ In New Zealand, ANTT has been used in some clinical and educational settings; however, a national framework, the New Zealand Aseptic Technique (NZAT) approach, has more recently been developed.^30^ It is worth noting that nearly half of the respondents were unsure whether their facility used a standardised aseptic technique framework (table 6). This is consistent with a study conducted by Gillespie *et al*^35^ who reported that, despite ANTT being embedded in some standards for a number of years, over half of the participants (n=59, 50.4%) reported being unaware of these standards. Participants in this study (n=236) did indicate that having a standardised technique is important to them (table 2). This supports findings in previous studies^23 36^ where nurses felt it was important to have a standardised approach to procedures requiring asepsis, and also stated that it positively impacted their practice. Uncertainty regarding framework adoption reduces the capacity of organisations to implement, evaluate and improve aseptic practice in a systematic way.

Surprisingly, a large number (n=174) of participants reported experiencing or observing confusion and incorrect practice of aseptic technique. A common theme was a lack of clarity around principles and terminology. One participant explained, *“It’s often confusing with terminology … clean, aseptic, sterile, different staff interpret them differently”* (P168), while another observed, *“There is a misunderstanding around what is a key or critical part” (P48).* Knowledge gaps were also evident in reports of poor practice*: “Colleagues donning gloves and touching everything from tray to the patient and everything in between, breaking the sterile field” (P231) and “Staff with sterile gloves on touching nonsterile items thinking they’re sterile due to the gloves being sterile” (P8) ([Table T5]*table 5). This is consistent with Gould *et al*,^23^ who also reported suboptimal practice of nurses’ aseptic technique.

While definitions exist, they are often inconsistent and mainly appear in policies or guidelines, with little reference to where the definition or principle originated from in empirical literature.^16^ Inconsistent terminology surrounding aseptic technique has been reported in empirical literature, creating challenges for both educators and clinicians in developing standardised teaching materials and policies.^24^ Previous studies^14 15^ have similarly highlighted a lack of clarification and standardisation in practice, with confusion particularly evident around the concept of sterility and cleanliness. Chen *et al*^37^ echoed these findings, noting that confusion between practical terms (eg, clean and sterile), imprecise definitions and the absence of unified standards. This is also evident in the findings of this study where there were notable misunderstandings and knowledge gaps around principles and what constitutes a clean, aseptic and sterile technique (online supplemental table 6).

Despite advances in hand hygiene and antimicrobial stewardship, aseptic technique remains a comparatively less developed component of infection prevention practice.^14 23 24^ This may be due to aseptic technique often being treated as a foundational skill embedded within local policies and task-based guidance rather than being emphasised as a set of underlying principles. It is typically taught during undergraduate nursing education and subsequently regarded as an ‘assumed’ skill, frequently learnt through rote practice in relation to other clinical skills rather than through conceptual understanding.^24^

Although aseptic technique originated in the controlled environment of the operating theatre, where research is well established, its application has expanded to ward-based settings and many routine nursing procedures with comparatively limited empirical evidence. At a system and governance level, the absence of standardised definitions and underpinning principles may constrain consistent approaches to competency assessment, reassessment and ongoing monitoring of aseptic technique.^16^ In the absence of clear or current understanding, nurses may experience frustration and confusion due to the inconsistent expectations across facilities and clinical contexts, and without clear or current understanding may be uncertain when an aseptic field has been compromised or may default to habitual practice, contributing to unwarranted variability in practice and potential patient safety risks.

Establishing a consensus definition of aseptic technique and related underlying principles is essential. This consensus would have the potential to be embedded within policies, procedures and educational resources to support the development of unified standards and improve the consistency of aseptic practice across clinical settings. These findings may translate into improvement aims for organisations focused on strengthening nurses’ understanding of aseptic technique principles, clarifying framework use and improving routine competency reassessment.

### Implications for improvement

The findings from this study indicate a need for system-level changes to strengthen the consistency and quality of aseptic technique. Drawing on the proposed contributing factors identified (figure 1), several practical improvement priorities can be considered.

Establishing standardised, evidence-based definitions and principles of aseptic technique is a foundational step; without these, organisations may lack a consistent basis for education, competency assessment and clinical governance.

Strengthening governance of competency reassessment is also needed, given that over half of participants had not been reassessed in the past 5 years. Progress in this area could be monitored by tracking the proportion of staff who complete reassessment within a defined timeframe, alongside evaluating whether compliance with aseptic technique improves in clinical audits.

Education in aseptic technique should be delivered as standard work, with emphasis on underlying principles. For example, organisations could introduce a brief annual competency module to address this. These suggested priorities are consistent with a quality-improvement approach and may provide organisations with a basis for guiding targeted changes and evaluating their impact over time.

### Limitations

This study has limitations that should be considered when interpreting the findings. The cross-sectional design does not allow for conclusions about causality. The recruitment approach may have led to selection bias toward nurses with a pre-existing interest in aseptic technique or infection prevention. Although several questions were adapted from a previous survey, and our survey was piloted, some items could still lead participants to a particular response. Furthermore, although several survey items were developed by subject experts, previously piloted and published in research, they were not formally psychometrically validated, which should be considered when interpreting the findings. Despite the survey being anonymous, the possibility of response bias cannot be discounted. It was not possible to determine an appropriate sample size estimate prior to study commencement, as the population undertaking aseptic technique is diverse and not fully understood or documented. Recruitment was conducted through open channels such as professional organisations, therefore, a denominator could not be established, and a response rate could not be calculated. As a result, the sample may not be representative of all nurses in these settings, thereby limiting the generalisability of these results. However, our response rate is consistent with other published Australian and New Zealand nursing surveys.^20^,^22^

### Contribution to the field and implications for further research

Our data support those of other studies that have demonstrated the scope to improve knowledge of aseptic technique, particularly the principles that guide practice and the need for a contemporary approach.^13 23 32 38^ This study further highlights the critical need to establish clear, practical terminology and principles that underpin aseptic technique, particularly within the clinical context of Australia and New Zealand. Establishing standardised terminology and principles, supported by evidence-based approaches, would better support the development of policies, education, and, ultimately, clinical practice, potentially reducing the risk of HAIs.

While this study focused on nursing practice, aseptic technique is relevant across multiple clinical disciplines. These findings may therefore influence broader improvement priorities, including international standardised definitions, shared competency frameworks and consistent approaches to education and assessments across clinical and educational settings.

Furthermore, this study identifies several important next steps, including a Delphi process involving key stakeholders, implementation and evaluation of a standards framework at a system or governance level, and the development of a minimum dataset to support consistent competency assessment and reassessment of aseptic technique. Collectively, these steps would promote a more unified approach to aseptic technique, minimise variation in practice and ultimately support patient safety.

## Supplementary material

10.1136/bmjoq-2025-003921 online supplemental file 1

## Data Availability

Data are available upon reasonable request.
