# Supplementary material for: Australian and New Zealand nurses’ understanding and application of aseptic technique in clinical contexts: a cross-sectional mixed-method study
Source: BMJ Open Qual. 2026 Jun 30;15(2):e003921. doi: 10.1136/bmjoq-2025-003921 (PMC13331115; doi:10.1136/bmjoq-2025-003921)
Supplement: online supplemental file 1 [file bmjoq-15-2-s001.docx]

# Australian and New Zealand nurses' understanding and application of aseptic technique in clinical contexts: A cross-sectional mixed-method study

## Supplementary material

#### Table S1- Definitions box

| Asepsis | Free from pathogenic micro-organisms in sufficient quantity to cause an infection [1] |
| --- | --- |
| Aseptic Technique | Is a set of practices that minimises the risk of introducing harmful microorganisms into wounds or vulnerable sites, while also preventing the transmission of pathogens from these areas to other individuals, including patients and healthcare staff [2, 3] |
| Principles | Fundamental concepts that guide decision-making and behaviour to minimise the risk of microbial contamination during clinical procedures, regardless of the specific task, setting, or technique used[4]. |
| Framework | Structured approach that organises concepts, principles, and practices to guide consistent application, education, and evaluation of clinical care across settings. |
| ANTT® | Clinical framework that supports aseptic technique by minimising contamination through non-touch principles and protection of key parts and key sites[5]. |

#### Table S2: Participant specialty **(n= 328)**

| ***Participant speciality***  Aged Care  Cardiology  Coronary Care  Emergency Care  Gastroenterology  General Medical  Intensive Care  Infection Prevention  Maternity  Mental Health  Mixed Surgical  Neurology  Oncology  Orthopaedic  Operating Theatres  Paediatrics  Primary Health  Renal  Radiology  Research  Rehabilitation Ward  Tertiary Education  Vocation Education and Training (VET)  Other | 27 (8.2)  7 (2.1)  2 (.6)  35 (10.7)  3 (.9)  28 (8.5)  18 (5.5)  42 (12.8)  10 (3)  7 (2.1)  13 (4)  1 (.3)  4 (1.2)  3 (.9)  25 (7.6)  17 (5.1)  24 (7.3)  5 (1.5)  3 (.9)  1 (.3)  6 (1.8)  37 (11.3)  2 (.6)  8 (2.4) |
| --- | --- |

#### Table S3: Education

What resources do you use to update your knowledge? (**n= 246**)

| **Topic**^ | **n** |
| --- | --- |
| Reviewing practice guidelines | 159 |
| Accessing protocols within my organisation | 135 |
| Through E-learning provided by my organisation | 176 |
| Other* | 68 |

^Respondents could select multiple barriers

*See S8 for other responses

#### Table S4: Educational approaches

What information or topics would you like covered in educational material or approaches? (Ranking question) (**n = 246**)

| **Topic** | **1** | **2** | **3** | **4** | **5** | **6** |
| --- | --- | --- | --- | --- | --- | --- |
| Practical elements of aseptic technique | 69 (28) | 54 (22) | 77 (31.3) | 38 (15.4) | 6 (2.4) | 2 (.8) |
| Importance of aseptic technique in infection prevention | 95 (38.6) | 54 (22) | 33 (13.4) | 33 (13.4) | 23 (9.3) | 8 (3.3) |
| Underlying principles of aseptic technique | 33 (13.4) | 79 (32.1) | 58 (23.6) | 42 (17.1) | 23 (9.3) | 11 (4.5) |
| Visual aids depicting aseptic technique | 23 (9.3) | 31 (12.6) | 39 (15.9) | 88 (35.8) | 54 (22) | 11 (4.5) |
| Data on infection rates related to aseptic technique | 21 (8.5) | 25 (10.2) | 27 (11) | 28 (11.4) | 96 (39) | 49 (19.9) |
| Common questions and answers | 5 (2) | 3 (1.2) | 12 (4.9) | 17 (6.9) | 44 (17.9) | 165 (67.1) |

#### Table S5: Understanding of aseptic technique (**n = 254**)

| **Category /Themes** | **Exemplars** |
| --- | --- |
| **Perception of purpose**  *Participants generally agreed that aseptic technique is a set of practices that prevents infection, but differed on who is being protected, the patient, the healthcare worker, or both.* | “A set of practices that prevent the spread of infections”  “Practice that protects the spread of organisms that cause infections”  “A set of principles and procedures which reduces the risk of introducing pathogens”  “Aseptic technique consists of practices designed to prevent healthcare-associated infections”  “Protect patient and nurse from infection, using sterile non-touch technique”  “Practices that protect workers”  “Practising to ensure patients are protected from healthcare-associated infections” “A practice that benefits and protects the patient from any hospital-acquired infections and also protects me from any bodily fluids, etc.” |
| **Confusion between clean and sterile**  *Several participants misunderstood or mixed up the concepts of “clean”, “sterile”, and “aseptic”.* | “Aseptic, meaning a technique of attending procedures that is deemed clean but not sterile.”  “The maintenance of a sterile procedure and free from microorganisms causing either infections or disease”  “Without sepsis”  “Clean, sterile field to work within.”  “Non-touch technique - opening supplies onto a sterile field and doing your best to keep it as clean as possible”  “Aseptic, meaning a technique of attending procedures that is deemed clean but not sterile.”  “Clean technique that prevents/decreases the risk of transfer of microbes from patient to staff and staff to patient during dressings/equipment change, etc”  “Establishing and maintaining a relatively sterile field throughout procedures”  “Aseptic: no pathogens, completely 'clean'/sterilised or as close to that standard as technologically capable.”  “It is between clean and sterile. it means free from germs.” |
| **Inconsistent definitions**  *While many participants attempted to define aseptic technique, many descriptions frequently emphasised the method itself rather than the overarching purpose.* | “Handwashing and non-touch during clinical procedures”  “No touch aseptic technique using sterile instruments & field.”  “A process to protect patients from hospital-acquired infections and provide PPE for staff when exposed to blood and body fluids - Standard Precautions”  “Methodical procedure providing care to key parts and key sites in a non-touch way” “Keeping everything that is sterile and only touching the bare minimum of the item so that anything that needs to go near the patient or into the patient remains sterile”  “Using my dominant hand as my clean hand and my other hand as dirty - I.e. the hand I would use to wipe down a wound. One swipe from cleaning to dirtiest, then discarding the swab. If required to get other supplies during procedure, I would ask for assistance or get myself & rewash my hands correctly/or with handwashing as supplied by my employer.”  “Aseptic technique is working with a sterile field and instruments with sterile gloves. As opposed to a medically clean procedure with sterile equipment, instruments, and dressings, but not using sterile gloves.” |

#### Table S6: Principles of aseptic technique (**n = 253**)

| **Category /Themes** | **Exemplars** |
| --- | --- |
| **Procedural focus over principles**  *Many participants described actions or checklists instead of articulating core principles.* | “Prepare all equipment. Perform hand hygiene. Utilisation of clinically clean or sterile equipment, Maintain sterile field, Utilise hands off / no touch technique”  “Basic hand hygiene, using either sterile or non-sterile gloves and using tweezers (or other instruments) to complete the procedure. It involves not applying the contaminated instruments in the sterile area. E.g., having a dirty and clean hand for wound dressings, pics and ports, etc., using your clean hand and instruments to pass to your dirty hand or instrument, to perform the task.  “No touch technique, one 'clean hand', one 'dirty hand'. Only touch the site once with gauze, one you lift up you need to get rid of it.” “Hand washing, Use of sterile gloves, keeping clean areas clean, and not contaminating a sterile field”  “PPE Hand hygiene Disposable of correct waste in correct bins Maintenance of aseptic technique Never contaminate key parts Cleaning the trolley down properly before and after use” |
| **Confusion between clean, sterile and aseptic fields**  *Participants frequently used the terms interchangeably or gave inconsistent definitions.* | “Clean field, Maintain clean field, Clean hands, Gloves if needed, Don’t touch key parts”  “The use of sterile clean equipment”  “Clean sterile area to work from, non-touch technique to reduce risk of infection and improve healing conditions.”  “For key parts and key sites to keep clean and stay clean”  “- Hand hygiene - Appropriate PPE - Maintaining the aseptic fields - minor or major - Environmental Controls where available and appropriate to the procedure ”  “Clean hands (soap and water wash + gloves), keeping all parts within a sterile area (eg dressing field), nothing is put onto the sterile area that is dirty” “Maintaining cleanliness or sterility of high touch points where applicable (e.g. items in a sterile pack while attending a wound dressing)”  “It is not a sterile procedure. It should be an efficient procedure. Used for fairly simple procedures.” |
| **Inconsistent principles**  *The number and content of principles listed varied widely (from one item to extensive checklists).* | “Not introduce any new pathogens”  “key parts - key sites”  “Hand washing, Use of sterile gloves, Keeping clean areas clean and not contaminating a sterile field”  “Infection Prevention: Removing infection from wound, Cleaning the wound”  “Hand Hygiene - Correct PPE Usage”  “Sterile hand hygiene (5 moments) key parts clean/disinfected, kidney dish, use, disposal of no reusable items, PPE”  “Hand hygiene, PPE, ANTT, proper waste management, proper sharps disposal.”  “Hand hygiene, PPE, sterile equipment, Aseptic field / maintaining sterile field, Non-touch technique”  “Reducing the risk of introduction of infection, Minimising risk of contamination from the environment, Minimising time spent on a procedure, Use of PPE & hand hygiene” “5 principles: No touch technique & maintaining aseptic field, Environmental control, Hand hygiene, PPE, Sequencing and risk assessment”  “Performing correct hand hygiene procedures, performing appropriate risk assessments prior to commencing the procedure, Ensuring barriers are used, and ensuring sterility of the packaging and equipment is complied with” |

#### Table S7: Frameworks

**What framework does your facility use to guide procedures requiring aseptic technique?** *(Open ended –Content analysis/ Word frequency?)* (**n=112**)

| **Category** | **Frequency** |
| --- | --- |
| ANTT ® | 32 |
| Local Policy | 21 |
| NSQHS | 8 |
| NZAT | 7 |
| State policy | 4 |
| NHMRC | 2 |
| ACORN | 3 |
| Textbook | 4 |
| Other | 10 |
| N/A | 8 |

#### Tables S8: ‘Other’ responses

**What resources did you use to update your knowledge? (OTHER)**

(**n=68**)

| **Category** | **Frequency** |
| --- | --- |
| Research articles | 13 |
| Professional bodies | 9 |
| External e-learning | 5 |
| Annual competencies | 4 |
| Policy development | 4 |
| Postgraduate studies | 4 |
| NHMRC | 3 |
| ANTT resources | 3 |
| ACQHS | 3 |
| Educating others | 2 |
| Inservice’s | 2 |
| Local policies | 1 |

**What are your barriers to performing aseptic technique? (OTHER)**

(**n =75**)

| **Category** | **Frequency** |
| --- | --- |
| Environmental factors | 11 |
| Staff culture | 9 |
| Emergencies | 5 |
| Knowledge gaps | 4 |
| Variance in training | 3 |
| Complex procedures | 2 |
| Other | 4 |
| N/A/ No barriers | 38 |

#### References

[1]. Chen Y, Cao X, Liu C, Sheng W, Wang J, Zhao L. Examining the knowledge level of nurses regarding aseptic non touch technique in nurses: a cross-sectional study. BMC Nursing. 2024;23(1):1-8. doi: 10.1186/s12912-024-02154-x.

[2]. Shaban RZ, Mitchell BG, Russo PL, Macbeth D. Healthcare-associated infections in Australia : Principles and practice of infection prevention and control. Chatswood, NSW: Elsevier; 2024.

[3]. NHMRC. Australian Guidelines for the Prevention and Control of Infection in Healthcare. In: Government A, editor. Canberra: Commonwealth of Australia: National Health and Medical Research Council; 2019.

[4]. Gould DJ, Chudleigh J, Purssell E, Hawker C, Gaze S, James D, et al. Survey to explore understanding of the principles of aseptic technique: Qualitative content analysis with descriptive analysis of confidence and training. American journal of infection control. 2018;46(4):393-6. doi: 10.1016/j.ajic.2017.10.008.

[5]. Clare S, Rowley S. Implementing the Aseptic Non Touch Technique (ANTT®) clinical practice framework for aseptic technique: a pragmatic evaluation using a mixed methods approach in two London hospitals. J Infect Prev. 2018;19(1):6-15. Epub 20170804. doi: 10.1177/1757177417720996. PubMed PMID: 29317909; PubMed Central PMCID: PMC5753945.
